# Localisation humérale d'une tumeur à cellules géantes récidivantes (à propos d'un cas)

**DOI:** 10.11604/pamj.2015.20.12.5519

**Published:** 2015-01-05

**Authors:** Youssef Nader, Issam Serghini, Idrissi Khalid Koulali, Hicham Salahi, Farid Galwia

**Affiliations:** 1 Pôle de Traumatologie et Orthopédie, Hôpital Militaire Avicenne, Marrakech, Maroc; 2 Pôle d'Anesthésie-Réanimation, Hôpital Militaire Avicenne, Marrakech, Maroc

**Keywords:** Cas rare, interessant, recidivant, agressif, difficulté therapeutique, rare case, interesting, recurrent, aggressive, therapeutic difficulty

## Abstract

Les auteurs rapportent un cas de localisation rare d'une tumeur à cellules géantes au niveau de la palette humérale du coude droit chez un militaire de 36 ans de sexe masculin, la radio standard montrait une image kystique ne soufflant pas la corticale. L'examen anatomo-pathologique a permis d’ établir le diagnostic et le traitement a fait appel: au début a une Exérèse chirurgicale totale et une greffe osseuse par un greffon iliaque de la totalité de la palette huméral qui s'est compliquée à 6 mois de recule d une récidive locale.

## Introduction

Les tumeurs à cellules géantes sont des tumeurs fréquentes; de comportement souvent déroutant, très récidivantes souvent bénignes et se localisant, la plus part du temps, aux extrémités des os longs des membres. La localisation distale de l'humérus est une entité rare. Leur traitement est presque exclusivement chirurgical mais non univoque. A propos d'un cas de localisation huméral exclusive, traitée chirurgicalement par résection et autogreffe, les auteurs insistent sur la rareté de cette localisation et sur la fréquence de ses récidives et proposent d'en faire une revue de littérature.

## Patient et observation

**Figure 1:**
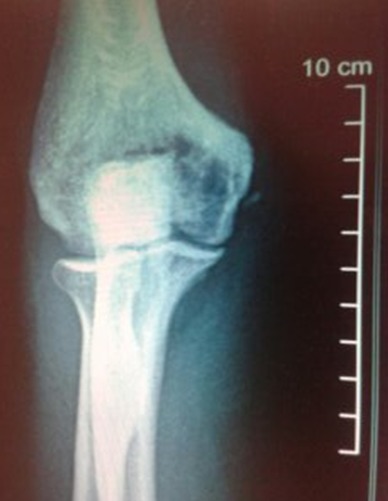
RX standards: tumeur a cellules géantes

Un patient âgé de 36 ans, de sexe masculin, militaire, sans antécédents pathologiques particuliers, consulte pour une tuméfaction de la face dorsale du coude droit, évoluant depuis un an et augmentant progressivement de volume, elle est douloureuse à la palpation, sans signes inflammatoires locaux, ni signes compressifs vasculaires ou nerveux. La radio standard montrait une image kystique ostéolytique pure, soufflant les corticales à bords flous, sans envahissement des parties molles (Figure 1). Le bilan biologique était sans particularité. Les signes cliniques, l'aspect radiologique et la localisation de la tumeur oriente vers le diagnostic de Chondrome. L'examen anatomo-pathologique après abord chirurgical postérieur électif a permis de poser le diagnostic de tumeur à cellules géantes. Le traitement consistait en un curetage de la tumeur qui avait détruit presque la totalité de la palette humérale, associé à une greffe osseuse corticospongieuse comblant le vide sur la totalité de l’épiphyse distale de l'humérus. Le greffon est prélevé au dépend de la crête iliaque homo latérale. L évolution après un recul de six mois était marqué par une récidive locale.

## Discussion

Les tumeurs à cellules géantes ou tumeurs à myeloplax sont des lésions ostéolytiques; de siège habituellement épiphysaire. Elles sont constituées d'un double contingent cellulaire: cellules géantes et éléments mononuclées. Ce sont des tumeurs peu fréquentes représentant en général 5 à 10/% des tumeurs primitives des os. souvent bénignes, elles constituent 13 à 15% des tumeurs bénignes des os. Mais elles peuvent parfois être malignes et représentent 2 à 5% des tumeurs malignes primitives de l'os. Les tumeurs à cellules géantes malignes constituent environ 10% de l'ensemble des tumeurs myeloplax [1–3]. Elles surviennent le plus souvent avec légère prédominance féminine entre 20 à 40 ans sur un os préalablement normal. Elles se localisent préférentiellement aux extrémités des os longs des membres (80 à 90% des cas) prés du genou, loin du coude (avec 60% au niveau du genou) [1–3]. Les extrémités distales de l'humérus, du cubitus, du radius et du péroné sont de sièges rares. Les métacarpes les phalanges représentent une localisation très rare avec moins de 1% de l'ensemble des localisations. L'atteinte des os courts et du tronc est moins habituelle surtout pour la rotule, le calcanéum, les os du carpe et du tarse [4]. Enfin, les formes multiples sont très exceptionnelles avec moins de 1% [2, 3]. Le tableau découverte d une tuméfaction qui alerte le patient, comme le cas de notre patient, En fin les facteurs pathologiques inaugurales sont possibles [5–7]. Le bilan biologique est souvent normal, cependant un bilan phosphocalcique est demandé; pour écarter une hyperthyroïdie. Quand à l'imagerie, dans la plus part des cas; de bonnes radiographies standards suffisent au diagnostic et permettent d'entreprendre le traitement; L'image de base est celle d une ostéolyse qui peut être pure ou en nid d'abeilles selon l'agressivité de la tumeur [8]. Un examen tomodensitométrie ou une étude en résonance magnétique peuvent être utiles si l'on craint un envahissement des paries molles [8]. La scintigraphie n'est utile que dans le dépistage des récidives. L'artériographie embolisante s'avère nécessaire dans les localisations difficiles (rachis, sacrum). Devant une localisation habituelle, les radiographies doivent faire discuter: un chondroblastome; un kyste anévrysmal ou une localisation épiphysite des chondrosarcomes à cellules claires. Dans les autres localisations la discussion est plus difficile, mais en règle et dans tous les cas c'est la biopsie seule qui permettra, de trancher un diagnostic positif, de préciser les éléments d évolutivités de la tumeur et d'identifier éventuellement sarcome [3, 5, 6, 8]Cependant des pièges de diagnostic histologique peuvent toujours se poser et nécessitent dans certains cas, une approche morphologique par techniques [9]; (ultra structurales, histochimie analyses imminohistochimie, histoenzymologie et auto historadiographie: marquage par un anticorps monoclonal permettant d'isolé les phases actives du cycle cellulaire, exemple anticorps monoclonal Ki -67).

**Figure 2:**
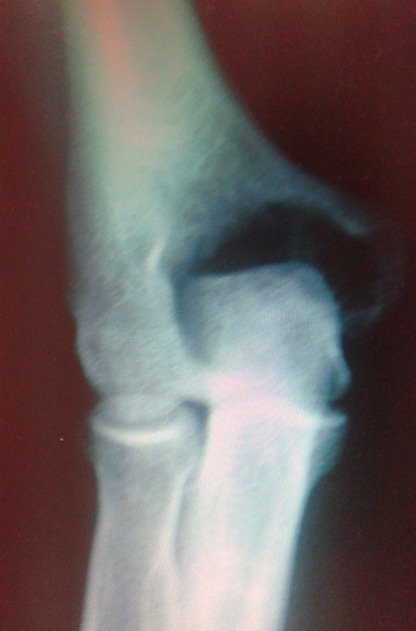
1^er^ récidive après curetage+greffe corticospongieuse

**Figure 3:**
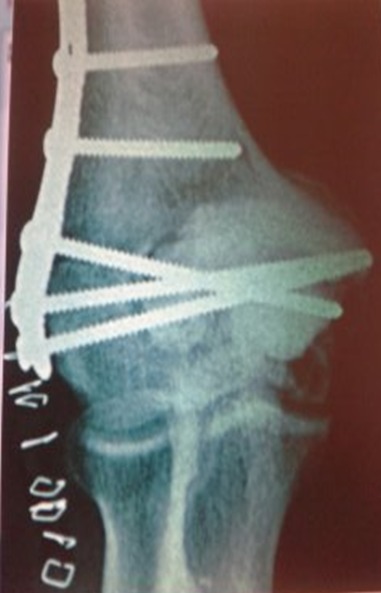
Traitement de la 1^ère^ récidive: curetage+comblement au ciment+ostéosynthèse

La classification histologique de 1940, de **Jaffé-Lichtenstein**[10] a un intérêt thérapeutique et pronostique, et propose le schéma suivant qui distinguent trois situations: **Grade1:** abondance de cellules géantes par rapport au contingent mononuclée, absence d'anomalies nucléaires à ce niveau, mitoses rares. Ce sont des tumeurs bénignes: histologiquement tranquilles. **Grade 2:** cellules mononuclées abondantes, discrètes anomalies nucléaires, activité mitotique marquée, mais sans formes atypiques. Ce sont tumeurs bénignes histologiquement actives **Grade 3:** c'est la cytologie et l'architecture d'un sarcome maligne. La TCG de notre patient est classé selon cette classification: **grade 2 Senerkin**, en1980 suggère une classification différente: **Degré 1:** il regroupe les TCG habituelles (grade 1, 2 de la classification: **JafféL-Lichtenstein**). **Degré 2:** ce sont des tumeurs borderline (grade 2 et plus). On y identifie des mitoses anormales **Degré 3:** ce sont des sarcomes avérés. D'autres classifications ont été proposées: celle de **Merle Aubigné**, et celle: **Campanacci**basées sur les aspects radiologiques et celle de **Enneking** [11] propose une autre qui prend en compte le comportement biologique global de la tumeur. L évolution de ces tumeurs est marqué par la fréquence des récidives (30%) qui peut être locales ou dans les parties molles. Elles surviennent dans les 3 premières années, mais sont possibles jusqu’à la 10^éme^ année [12]. Chez notre patient et après un recul de six mois nous avons détecté malheureusement une récidive locale (Figure 2). Cependant, une dégénérescence maligne peut également se voir. Elle peut être spontanée au fil des récidives ou radio induite. Mais elle peut également survenir sur des formes ou il existait dés le départ des zones malignes, parfois petites et ayant été méconnues lors de biopsies trop économiques [13]. D'ou l'intérêt de faire de la première biopsie un véritable temps de curetage-comblement, les récidives doivent également être rebiopsié avant de décider du nouveau traitement. Enfin des métastases pulmonaires bénignes peuvent également apparaître dans l’évolution d'une tumeur à cellules géantes bénigne. Le traitement est souvent chirurgical [14–18], intra lésionnel: avec curetage simple, curetage comblement classique par greffe osseuse; curetage comblement avec adjuvants (azotes liquide, eau distillée, phénol, ciment chirurgical); ou extra lésionnel: avec résection marginale et reconstruction, résection large ou amputation. Parfois d'autres procédés thérapeutiques peuvent être utilisées: embolisation, injection journalière intra tumorale de calcitonine; radiothérapie ou chimiothérapie. Les tumeurs malignes bénéficient, de 2 à 3 mois de chimiothérapie, suivi d'une résection reconstruction le plus souvent et très rarement d'une amputation; si envahissement des parties molles, avec reprise de chimiothérapie de 4 à 6 mois en postopératoire [15, 16, 18]. Les métastases pulmonaires bénignes sont réséquées par thoracotomie chaque fois que possible. Les tumeurs bénignes récidivantes font souvent l'objet d'une seconde biopsie de confirmation et d'un second curetage avec comblement adjuvante le plus souvent on utilise du ciment chirurgical, ce qui a été réalisé chez notre patient complété par une ostéosynthèse par une plaque lecéstre (Figure 3).

## Conclusion

La localisation humérale distale des tumeurs à cellules géantes et rares. L'aspect radiologique fait penser aux tumeurs bénignes: chondromes; chondroblastome. Le diagnostic positif est purement histologique et le traitement est chirurgical: résection-greffe ou résection-comblement en cas de récidive bénigne.
